# Effects of Hanwoo (Korean cattle) manure as organic fertilizer on plant growth, feed quality, and soil bacterial community

**DOI:** 10.3389/fpls.2023.1135947

**Published:** 2023-03-21

**Authors:** Junkyung Lee, Na-Yeon Jo, Su-Yeon Shim, Le Tran Yen Linh, Soo-Ryang Kim, Myung-Gyu Lee, Sun-Goo Hwang

**Affiliations:** ^1^ College of Life and Environment Science, Sangji University, Wonju-si, Republic of Korea; ^2^ Department of Smart-Farm, Sangji University, Wonju-si, Republic of Korea; ^3^ Department of Earth Environment Engineering, Sangji University, Wonju-si, Republic of Korea

**Keywords:** organic fertilizer, bacterial community, amplicon sequence variant, Hanwoo manure, maize

## Abstract

**Introduction:**

The development of organic manure from livestock excreta is a useful source for sustainable crop production in environment-friendly agriculture. Organic manure increases soil microbial activity and organic matter (OM) supply. The excessive use of chemical fertilizers (CFs) leads to air and water pollution caused by toxic chemicals and gases, and soil quality degradation via nutrient imbalance due to supplying specific chemical components. Thus, the use of organic manure will serve as a long-term supply of various nutrients in soil *via* OM decomposition reaction as well as the maintenance of environment.

**Methods:**

In this study, we aimed to analyze the diverse effects of Hanwoo manure (HM) on plant growth, feed quality, and soil bacterial communities in comparison with CFs, commercial poultry manure (CM), and the combined use of chemical fertilizer and Hanwoo manure (HM+CF). We analyzed the contents of crude matter (protein, fat, fiber, and ash), P, acid detergent fiber (ADF), and neutral detergent fiber (NDF) through feed quality analysis, and the contents or activities of total phenol, total flavonoid, ABTS, nitrite scavenging, and reducing power *via* the antioxidant assay. Furthermore, the soil microbial communities were determined using 16S rRNA sequencing. We compared the soil bacteria among different soil samples by using amplicon sequence variant (ASV) analysis.

**Results and discussion:**

We observed increased OM in the soil of the HM group compared to that of the CF and non-treated groups over a period of two years. Moreover, HM+CF treatment enormously improved plant growth. Organic manure, especially HM, caused an increase in the content of crude ash and phosphorus in plants. There were no significant differences in total polyphenol, total flavonoid, ABTS, nitrite scavenging, and reducing power in plants between HM and CF groups. Finally, we detected 13 soil bacteria (*Acidibacter*, *Algisphaera*, *Cystobacter*, *Microvirga*, *Ohtaekwangia*, *Panacagrimonas*, *Pseudarthrobacter*, *Reryanella*, *Rhodoligotrophos*, *Solirubrobacter*, *Stenotrophobacter*, *Tellurimicrobium*, and *Thermomarinilinea*) that were considerably correlated with OM and available phosphorus, and three considerably correlated bacteria were specifically distributed in CF or organic manure. The results suggest that HM is a valuable source of organic manure that can replace CF for sustainable crop production.

## 1 Introduction

Chemical fertilizers (CFs) are mainly used to improve crop production and plant growth because they are easily absorbed by plants (Savci, 2012; Han et al., 2016). Using these CFs leads to nutritional imbalances and acidification in the soil because of the high accumulation of several chemical components (Simpson et al., 2011; Bisht and Chauhan, 2020). Although organic manure has low nutrient contents and decomposes nutrients slowly, it has various benefits in the soil, such as a good balance of multiple nutrients and an improvement in microbial activity and physical soil structure (Han et al., 2016). In previous studies, long-term use of organic fertilizers increased crop yield and nutrient uptake rate compared to that of CFs (Lu et al., 2021; Nie et al., 2007). Furthermore, Ji et al. (2017) reported that liquid fertilizer from organic matter (OM) improved root development and soil microbial diversity in *Chrysanthemum morifolium*. Thus, organic liquid fertilizer can suitably decrease the use of CFs in agriculture owing to their positive effects on plant growth and soil improvement.

Many microorganisms exist in the soil, including fungi, viruses, and bacteria. Microbial communities in the soil are influenced by the growth of different plant species (Fierer, 2017). The microbial communities affected water and nutrient uptake from the environment, leading to plant growth promotion (Kim et al., 2012). The plant growth-promoting bacteria (PGPB) play a role in the nutrient fixation of nitrogen, iron, and phosphorus depending on soil composition; plant growth is influenced by providing the fixed nutrients (Kim et al., 2012). Moreover, these beneficial bacteria positively affected organic matter decomposition and soil structure maintenance (Mumtaz et al., 2017; Suman et al., 2022). The proper use of PGPB can reduce the application of chemical fertilizers in agriculture (Adesemoye et al., 2009). The physical and chemical properties and soil microbiome were improved by providing organic manure (Adekambi and Drancourt, 2004; Naik et al., 2019). In a multi-generation experiment, the soil microbial community influenced the flowering time of *Arabidopsis thaliana* (Panke-Buisse et al., 2015). Furthermore, the plant pathogens are suppressed by soil microbiomes, such as *Pseudomonas* and *Bacillus* bacteria, which can be subsequently transferred by soil transplantation (Wei et al., 2019). In a previous study, organic biofertilizer inoculated with *Trichoderma* improved the production and quality of tomatoes compared with CF (Ye et al., 2020). Thus, the proper use of organic manure may reduce CF usage for environmental conservation in agriculture.

Maize (*Zea mays* L.) is an important crop worldwide, with an abundance of nutritional benefits and fibrous matter as an edible crop for humans and livestock (Duvick, 2005; Zhang et al., 2022). Corn has high digestibility in livestock diets because of the different types and associations with corn starch (Loy and Lundy, 2019). The livestock’s rumen can digest corn with a soft texture. Corn is relatively high in sulfur-containing amino acids, such as methionine and cysteine, while it is low in essential amino acids, such as lysine and tryptophan (Loy and Lundy, 2019). However, corn has been shown to improve protein quality and oil and amylose contents, depending on human requirements (Darrah et al., 2019). Unlike white corn, the kernels of yellow corn have carotenoid pigments; thus, yellow corn silage can be a useful source of provitamin A (Watson, 1962). The high oil content of corn results in a high level of energy supply in poultry (Lee et al., 2001) and swine livestock (Adeola and Bajjalieh, 1997). Corn contains diverse types of B vitamins, such as niacin, biotin, thiamin, and pyridoxine, but not cobalamin and vitamin E (Ball and Ratcliff, 1978; Cort et al., 1983; Lynch et al., 1996; Loy and Lundy, 2019). Thus, corn provides high amounts of energy and nutrients to livestock. Therefore, the demand for maize has rapidly increased worldwide, especially in developing countries (Shiferaw et al., 2011).

Cattle manure contains relatively lower nitrogen (N) and phosphorus pentoxide than poultry, swine, and sheep (Cravotta, 1995). In particular, the N content is most deficient in cattle manure, whereas it is highest in poultry manure based on unit weight. However, manures from dairy cows and beef cattle have higher contents of dry materials as OM sources than those from hog and poultry. As the economy grows, human demand for meat increases and excessive supply has increased environmental and social problems (Tilman et al., 2011). The amount of livestock excretions in Hanwoo cattle in Korea is higher than that in swine and poultry (Choi, 2007). Although Hanwoo manure (HM) has a low N content, the use of HM must be encouraged in crop production, which will contribute to environmental conservation and environment-friendly agricultural development. In this study, we attempted to observe the diverse effects of HM on plant growth, antioxidants, and microbial communities in the cultivation of forage maize and compare its impact with CF and commercial poultry manure. Furthermore, the availability of HM was evaluated to reduce the use of chemical fertilizer in the forage maize production.

## 2 Materials and methods

### 2.1 Plant growth condition and survey

Maize (*Z. mays* L., Kwangpyeongok) was obtained from the Agricultural Technology Center (Hoengseong-gun, Gangwon-do, Republic of Korea), and its seeds were grown in a greenhouse for two weeks. The seedlings were transplanted into an experimental field (37° 22’ 16.2″ N, 127° 55’ 30.1″ E) at Sangji University (Wonju-si, Gangwon-do, Republic of Korea). The experiment for non-treatment (NT), CF, commercial manure (CM), HM, and a mixture of HM and CF was performed in the field using randomized complete block design. Thus, all treatments were randomly assigned to the experimental units within the blocks in the experimental field. Fertilizers were applied following the cultivation method used in a previous experiment by Byeon et al. (2022). Plant growth was observed with respect to plant length, leaf width, and leaf length in the different fertilizer-treated plant groups. The chlorophyll concentration of the leaves was measured using a SPAD-502plus chlorophyll meter (Konica Minolta Inc., Teban Gardens Crescent, Singapore). Fresh and dry weights were measured in the aboveground parts of the plants before and after dehydration, respectively. The plant tissues were dried at 60°C for 24 h.

### 2.2 Total nitrogen

A soil sample of 10 g was mixed with 50 mL of distilled water; then, the electrical conductivity (EC) and pH were detected using multiparameter analysis (Edge HI2020, HANNA instruments, Woonsocket, USA). The total nitrogen was measured using the Kjeldahl method (Barbano et al., 1990; Aoac, 1995), where a 5 g sample was mixed with a solution of 50 g potassium sulfate and 50 g copper (II) sulfate (9:1). The samples were heated for 4 h. Subsequently, 500 µL of phenolphthalein solution was added (0.04 g phenolphthalein, 50 mL 95% ethyl alcohol, and 50 mL distilled water). Sodium hydroxide (NaOH; 0.01 N) was added to the prepared sample until it turned red. To collect the distillate from the sample, 2% bromocresol green solution (0.5 g bromocresol green, 0.1 g methyl red, and 100 mL 95% ethyl alcohol) was used. Finally, the end-effect points of 0.01 N sulfuric acid (H_2_SO_4_) for changing color from blue to red were measured. The total nitrogen content was calculated for each treatment as follows:


$$factor\;of\;0.01N\;\text{H}2\text{SO}4\;=\frac{amounts\;of\;0.1N\;NaOH\;for\;the\;end-effect\;point\;\times \;f\;of\;0.1N\;NaOH}{amounts\;of\;0.1N\;\text{H}2\text{SO}4}$$

$$Total\;nitrogen\;\left(\%\right)=\frac{\left(T-B\right)\;\times \;f\;\times \;Normality\;\;\times \;\;14\;\;\times \;\;100}{sample\;weight\;\left(mg\right)}\times \;\;diluted\;ratio\;of\;sample$$

where T represents the amounts of 0.01N H_2_SO_4_ for the end-effect point, B represents the blank, f represents the factor of 0.01N H_2_SO_4_, and Normality represents the concentration (N) of H_2_SO_4_.

### 2.3 Total phosphorus

Total phosphorus (P) and available phosphorus pentoxide (P_2_O_5_) were detected using the Lancaster soil testing method (Lancaster, 1980). A soil sample of 5 g was added into the 20 mL extracting solutions that comprised 400 mL acetic acid (CH_3_COOH), 300 mL 10N lactic acid (CH_3_CH[OH]COOH), 22.2 g ammonium bifluoride (NH_4_F), 133.3 g diazanium sulfate ([NH_4_]2SO_4_), and 170 g NaOH, and the mixture was boiled for 10 min. Standard P solutions (10, 100, and 1000 ppm) were used to evaluate the available P_2_O_5_ in the samples. A 6 mL ammonium paramolybdate solution (200 mL ammonium paramolybdate and 100 mL 0.8M boric acid [H_3_BO_3_]) was added for absorbance determination using an Ultraviolet-visible (UV-Vis) spectrophotometer (NEO–S2117, NEOGEN). The total P and available P_2_O_5_ were calculated for each treatment as follows:


$$Available\;phosphorus\;pentoxide=\;\frac{detected\;ppm\;\;\times \;\;extracted\;liquid\;\left(ml\right)}{sample\;weight\;\left(g\right)}\times \;\;2.2914$$

Total P = Available phosphorus pentoxide  ×  0.4364

### 2.4 Exchangeable cations

Exchangeable cations were detected using the ammonium acetate method. The four exchangeable cations (potassium [K], calcium [Ca], magnesium [Mg], and natrium [Na]) were determined in the 5 g samples and 50 mL of 1N-NH_4_OAc at pH 7 by inductively coupled plasma atomic emission spectrometry (ICP-OES; SPECTROBLUE, SPECTRO). The four exchangeable cations were calculated for each treatment as follows:


$$Ex-cations\;=\;ICP-AES\;value\;\times \;\frac{extracted\;liquid\;\left(mL\right)}{sample\;weight\;\left(g\right)\;\times 10}\;÷\;electrochemical\;equivalent$$

The electrochemical equivalent was 39.1 for K, 20.04 for Ca, 12.15 for Mg, and 22.99 for Na.

### 2.5 Organic matter

OM was determined using the Tyurin method (Schollenberger, 1927). The 5-g sample was added to 10 mL of 0.4 N potassium dichromate solution (40 g potassium dichromate [K_2_Cr2O_7_] per 1 L H_2_SO_4_). The solution was then boiled for 5 min on a hot plate (C-HP, CHANGSHIN SCIENCE), and 150 mL of distilled water was added. Five milliliters of 85% H_3_PO_4_ and 500 µL diphenylamine were added to the sample. To determine the end-effect point, 0.2N ammonium iron (II) sulfate hexahydrate solution (78.44 g [NH_4_]2SO_4_FeSO_4_ 6H_2_O per 1 L H2_S_O_4_) was added to the reactants until it turned from orange to green. The OM was calculated for each treatment as follows:


$$OM\;\left(\%\right)\;=\;\left(B\;–\;T\right)\;\;\times \;\;0.2\;\times \;3\;\times \;0.001\;\;\times \frac{100}{sample\;weight\;\left(g\right)}\;\times \;\;1.724$$

where T represents the amounts of 0.2N ammonium iron (II) sulfate hexahydrate solution for the end-effect point, B represents the blank, 0.2 represents the normal concentration of ammonium iron (II) sulfate hexahydrate solution, 3 represents the chemical equivalent of 1 mg carbon, and 1.724 represents the carbon ratio for soil erosion.

### 2.6 Cation exchange capacity of fertilizers

The cation exchange capacity (CEC) of the fertilizer was determined using the ammonium acetate method (Thomas, 1982) with 5 g of sample and 100 mL of 1N NH_4_OAc. After 4 h of incubation, 100 mL of 80% ethyl alcohol (pH 7.0) was added. Then, 500 µL phenolphthalein solution (0.04 g Phenolphthalein, 50 mL ethyl alcohol, and 50 mL distilled water) and MgO were added until they turned red. Finally, 20 g of H_3_BO_3_ and 5 mL of 2% bromocresol green solution per liter (L) were added to the reactants, and the endpoint of 0.01N H_2_SO_4_ for changing color from blue to red was measured. The CEC was calculated for each treatment as follows:


$$CEC\;\left(me/100g\right)\;=\;\frac{T-B}{sample\;weight\;\left(g\right)}\times \;f\;\times \;\;Normality\;\times \;\;100$$

where T represents the amounts of 0.01N H2_S_O_4_ at the end-effect point, B represents the blank, f represents the f of 0.01N H_2_SO_4_, and Normality represents the concentration (N) of H_2_SO_4_.

### 2.7 Crude protein content

The content of crude protein was extracted following the Kjeldahl method (Barbano et al., 1990; Aoac, 1995). Briefly, 0.5 g of a dried sample was mixed with 7 g of potassium sulfate solution (9 g potassium sulfate and 1 g copper sulfate) and 10 mL of H_2_SO_4_. Then, 100 µL liquid indicator was added to the prepared mix. The solution was then distilled after adding zinc and sodium hydroxide solution (sodium hydroxide 500 g, sodium thiosulfate 100 g, distilled water 1 L). The amount of 0.1 N hydrochloric acid was observed until the desired color (reddish brown) was obtained. The percentage of crude oil content was calculated using the following formula:


$$Crude\;protein\;\left(\%\right)\;=\;\frac{0.00140067\;\left(0.1N\;HCl\right)\;\times \;T\;\times \;F\;\times \;6.25\;\times \;100}{W}$$

where T represents the amount of 0.01 N H_2_SO_4_ for the end effect point, F represents the amount of 0.1 N HCl for the blank, and W represents the weight of the sample.

### 2.8 Crude fat content

The crude fat content was extracted using the ether extract method (Padmore, 1990). In this method, after drying 2 g of the sample on a filter paper for 2 h at 100°C, ether was mixed and placed in the Soxhlet extractor (ANKOM XT15 Extractor, ANKOM Technology, USA) for boiling at 80°C for 8 h to extract the fat. The ether was extracted and dried in the solvent flask for 3 h at 100°C. Afterward, the sample was cooled in a desiccator for 40 min, and using the following formula, the crude fat content was calculated:


$$Crude\;fat\;\left(\%\right)\;=\;\frac{a-b}{c}\;\times \;\;100$$

where a and b are the total mass of the solvent flask after and before extraction, respectively, and c is the total mass of the sample.

### 2.9 Crude fiber content

The content of crude fiber was extracted by following Henneberg and Stohmann’s method (Henneberg and Stohmann, 1859). In this method, a mix of 1 g of sample, 50 mL of 5% sulfuric acid solution (27 mL H_2_SO_4_, and 1 L distilled water), and 150 mL distilled water were placed in a 500 mL beaker, followed by the addition of 2 drops of an anti-foaming agent. After boiling the mix for 30 min, it was filtered, and the residue was washed with hot distilled water until the alkaline was eliminated with quantitative filter paper (2.5 µM). Then, 130 mL of the washed residue was added to 50 mL of 5% sodium hydroxide solution (50 g NaOH and 1 L distilled water) in a beaker, and distilled water was added until the total volume reached 200 mL. After boiling the mix again for 30 min and washing the residue with hot distilled water, the resultant residue was washed thrice with 95% ethyl alcohol and twice with ethyl-ether, followed by drying the residue for 2 h at 100°C and 2 h at 135°C. The dried mass was then cooled in a desiccator for 40 min, burned in a porcelain crucible, and cooled again. The crude fiber content was then calculated as follows;


$$Crude\;fiber\;\left(\%\right)\;=\;\frac{d-a}{s}\;\times \;\;100$$

where d represents the dry weight of the residue filtered after decomposition, a represents the residue after the last burning in the porcelain crucible, and s represents the weight of the sample.

### 2.10 Crude ash content

The crude ash content was determined following Liu’s method (Liu, 2019). In this method, the porcelain crucible was burnt in an electric furnace (Lindberg/Blue M, Thermo Fisher Scientific, USA) at 600°C for 1 h and cooled in a desiccator for 40 min. Then, 2 g of the sample was placed in an electric furnace at 600°C for 2 h, cooled in a desiccator for 40 min, and measured. The crude ash content was calculated for each treatment as follows:


$$Crude\;ash\;\left(\%\right)\;=\;\frac{a-b}{c}\;\times \;\;100$$

where a represents the weight of the burnt sample and the porcelain crucible, b represents the weight of the porcelain crucible, and c represents the raw sample weight.

### 2.11 Phosphorus content

The P content was measured following Cavell’s method (Cavell, 1955). In this method, the P content of corn was determined by measuring its absorbance after adding a coloring agent. First, 1 mL of sample solution (filtrate of 2 g burned sample mixed with 10 mL of hydrochloric acid [1:1]) was taken in a 25 mL mass flask and mixed with 2.5 mL of ammonium molybdite solution (25 g ammonium molybdite with 400 mL distilled water). The absorbance was then measured at 470 nm wavelength. The P content was calculated for each treatment as follows:


$$Phosphorus\;\left(\%\right)\;=\;\frac{a/b\;\;\times \;\;c}{d\;\;\times \;106}\;\times \;\;100$$

where a is the absorbance of the sample, b is the standard absorbance (1 ppm), c is the dilution factor, and d is the sample weight.

### 2.12 Neutral detergent fiber

Neutral detergent fiber (NDF) was measured using 1 g of sample and 100 mL of neutral detergent solution (150 g of sodium lauryl sulfate, 93.05 g of EDTA disodium salt, 34.05 g of sodium borate, 22.8 g of sodium phosphate, 22.8 g of dibasic dodecahydrate, and 50 mL of ethylene glycol monoethyl ether). Then, 5 L of distilled water was added to 2 mL of decahydronaphthalene and 0.5 g sodium sulfite. The mix was boiled for 1 h in an automated fiber analyzer (ANKOM A2000, ANKOM Technology, USA) and then filtered through a glass filter. The residue was then washed with acetone and dried at 105°C for 4 h. NDF was calculated for each treatment as follows:


$$NDF\;\left(\%\right)\;=\;\frac{a-b}{c}\;\times \;\;100$$

where a represents the sample weight after drying, b represents the residual weight, and c represents the sample weight.

### 2.13 Acid detergent fiber

For measurement of acid detergent fiber (ADF), 2 g of the sample was added to a 500 mL beaker along with 100 mL of acid detergent solution (melted mix of 20 g of cetyltrimethylammonium bromide [CTAB] and 1 N 1 L of H_2_SO_4_), and 2 mL of decalin. The mixture was then boiled for 1 h in an automated fiber analyzer and filtered using a glass filter. The residue was washed with acetone and dried at 105°C for 4 h. The ADF was calculated for each treatment as follows:


$$ADF\;\left(\%\right)=\;\frac{a-c}{c}\;\times \;\;100$$

where a represents the sample weight after drying, b represents the residual weight, and c represents the sample weight.

### 2.14 Antioxidant analysis

The top part of forage maize was used to evaluate the antioxidant activities of plants in the different fertilizer-treated groups. The total phenol and flavonoid contents, nitrate-scavenging activities, reducing power, and ABTS were determined as described previously (Jo et al., 2022). All measurements were performed in triplicate. The plant extracts were collected using methanol, and standard curves were generated for quantitative analysis using standard materials (quercetin for total flavonoid and gallic acid for total phenol) (**Figure S1**). A high R-squared value (R^2^ > 99) was obtained in the regression line. The absorbance was measured using a spectrometer at 760, 510, 520, 734, and 700 nm for total phenol, total flavonoid, nitrate-scavenging activity, ABTS assay, and reducing power assay, respectively.

### 2.15 Soil microbiome 16s rRNA sequencing analysis

To compare the microbial diversity in the fertilizer-treated soils, an Illumina MiSeq microbiome analysis was conducted. The soil microbial DNA was isolated using DNeasy Power Soil Kit (Qiagen, Hilden, Germany) followed the manufacturer’s protocol, and a DNA library was generated using the Illumina 16S Metagenomic Sequencing Library (Amplicon et al., 2013). According to the manufacturer’s instructions, polymerase chain reaction (PCR) was performed using Herculase II Fusion DNA Polymerase (Agilent Technologies, Santa Clara, CA, USA). The first PCR process involved heat activation for 30 min at 95°C, followed by 25 cycles of denaturation for 30 s at 95°C, annealing for 30 s at 55°C, extension for 30 s at 72°C, and a final extension for 10 min at 72°C. The sequences of the primer pairs used for the first amplification are as follows:

V3-F: 5′-TCGTCGGCAGCGTCAGATGTGTATAAGAGACAGCCTACGGGNGGCWGCAG-3′,
V4-R: 5′-GTCTCGTGGGCTCGGAGATGTGTATAAGAGACAGGACTACHVGGGTATCTAATCC-3′.

AMPure XP beads (Agencourt Bioscience, Beverly, MA, USA) were used to purify the first PCR products. The purified first PCR product was used for library construction using the NexteraXT Indexed Primer (Illumina Inc., CA, USA). The second PCR and purification were performed using the same method as the first PCR. Sequencing was performed using the MiSeq™ platform (Illumina, San Diego, CA, USA) with a paired-end method (2 × 300 bp).

### 2.16 Amplicon sequence variant and statistical analysis

Raw sequencing data (fastq) were used to remove adapter sequences using the Cutadapt program (Martin, 2011). We performed an error correction of filtered paired-end sequencing data using the R package DADA2 (Callahan et al., 2016) and identified the amplicon sequence variant (ASV). The ASVs obtained from different samples were normalized using QIME (Caporaso et al., 2010). The taxonomy of each ASV was identified from the NCBI 16S microbial database using BLAST+ with a query coverage of 85% (Camacho et al., 2009). Statistical analysis of bacterial diversity was performed using the R packages dplyr (Wickham et al., 2015), taxa (Foster et al., 2018), apes (Paradis et al., 2004), ggrepel (http://cran.nexr.com/web/packages/ggrepel/index.html), pyloseq (McmurdieHolmes, 2013), DESeq2 (Love et al., 2014), vegan (https://cran.r-project.org/web/packages/vegan/index.html), ggsignif (https://cran.r-project.org/web/packages/ggsignif/index.html), and ggplot2 (Wickham and Chang, 2016). Significant differences were evaluated using the R package Agricolae (https://cran.r-project.org/web/packages/agricolae/index.html) with Duncan’s test for comparisons between different treatment groups (p ≤ 0.05). To determine the significant correlation between soil chemical components and bacterial communities, the Mantel test was performed using the R package vegan (https://cran.r-project.org/web/packages/vegan/index.html) with 9,999 permutations, and canonical correspondence analysis (CCA) was performed using the R package CCA (https://cran.r-project.org/web/packages/CCA/index.html).

## 3 Results

### 3.1 Soil chemical components

We analyzed the chemical components of soil in each experimental field [NT, CF, CM, HM, and combined treatments of Hanwoo manure and chemical fertilizer (hereinafter named HM+CF)] after harvesting maize (**Table 1**). The pH was considerably lower in the CF group (5.51) than in the other groups (5.57 for NT, 6.21 for CM, 5.93 for HM, and 5.79 for HM+CF). The highest CEC was observed in CM (10.93 cmol+/kg). Total Nitrogen content was markedly higher in the CM (0.139%) and HM+CF (0.139%) groups; however, the HM group (0.131%) was not significantly different from the CM and HM+CF groups. The available P was increased in CM (301.70 mg/kg), CF (297.85 mg/kg), and HM+CF (285.86 mg/kg). Ca and Mg exchangeable cations were high in the CM (7.72 cmol+/kg for Ca, 1.37 cmol+/kg for Mg) and HM+CF (7.98 cmol+/kg for Ca, 1.38 cmol+/kg for Mg) groups, while exchangeable K and Na showed no considerable difference. Finally, a high OM was observed in HM (2.58%) and HM+CF (2.49%) groups. The EC and salinity showed no considerable differences among the different experimental soils. Overall, livestock manure showed high total nitrogen, available P, and OM contents in the soil, suggesting that the nitrogen and available P contents increased in the soil owing to increased OM.

**Table 1:** Chemical components of fertilizers remaining in the soil.

	NT	CM	HM	CF	HM + CF
pH[1:5]	5.57 (± 0.17) ab	6.21 (± 0.18) a	5.93 (± 0.13) ab	5.51 (± 0.14) b	5.79 (± 0.22) ab
EC[1:5] (dS/m)	0.10 (± 0.006) a	0.11 (± 0.008) a	0.10 (± 0.05) a	0.10 (± 0.008) a	0.12 (± 0.006) a
C.E.C. (cmol+/kg)	9.02 (± 0.17) b	10.63 (± 0.17) a	9.45 (± 0.15) b	9.24 (± 0.17) b	9.58 (± 0.13) b
Total N (%)	0.105 (± 0.004) c	0.139 (± 0.002) a	0.131 (± 0.003) ab	0.126 (± 0.004) b	0.139 (± 0.003) a
Available P (mg/kg)	201.63 (± 7.72) c	301.70 (± 4.54) a	245.32 (± 6.61) b	297.85 (± 5.56) a	285.86 (± 3.52) a
Exchangeable K (cmol+/kg)	0.07 (± 0.01) a	0.09 (± 0.01) a	0.11 (± 0.013) a	0.08 (± 0.01) a	0.09 (± 0.015) a
Exchangeable Ca (cmol+/kg)	7.32 (± 0.092) b	7.72 (± 0.093) a	7.07 (± 0.099) b	7.24 (± 0.085) b	7.98 (± 0.104) a
Exchangeable Mg (cmol+/kg)	1.20 (± 0.02) b	1.37 (± 0.03) a	1.26 (± 0.02) b	1.20 (± 0.02) b	1.38 (± 0.02) a
Exchangeable Na (cmol+/kg)	0.08 (± 0.01) a	0.07 (± 0.01) a	0.09 (± 0.01) a	0.08 (± 0.005) a	0.09 (± 0.01) a
Salinity (%)	0.005 (± 0.0008) a	0.004 (± 0.0005) a	0.005 (± 0.0005) a	0.005 (± 0.0005) a	0.005 (± 0.0005) a
OM (%)	1.48 (± 0.05) d	2.32 (± 0.03) b	2.58 (± 0.04) a	1.95 (± 0.03) c	2.49 (± 0.03) a

NT, non-treatment; CM, commercial manure; HM, Hanwoo manure; CF, chemical fertilizer; HM + CF, combined use of Hanwoo manure and chemical fertilizer.

Lowercase letters represent significant differences (p < 0.05) between groups as determined using Duncan’s test.

### 3.2 Effect of different liquid fertilizers on plant growth

**Figure 1:**
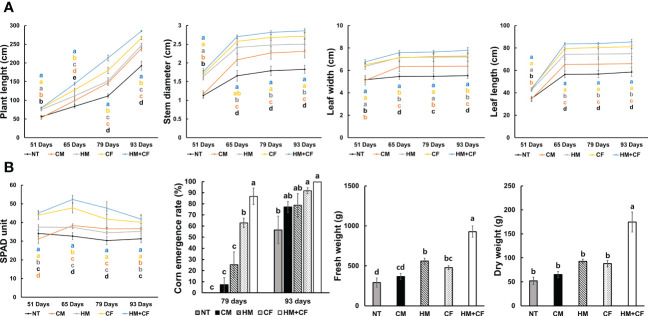
Difference in the growth of corn (NT, non-treatment; CM, commercial manure; HM, Hanwoo manure; CF, chemical fertilizer; HM+CF, combined use of Hanwoo manure and chemical fertilizer). **(A)** Plant length, stem diameter, and the width and length of leaves 93 days after planting. **(B)** SPAD unit, corn emergence rate, and fresh and dry weight of plant 93 days after planting. The line colors represent the different fertilizer treatments. The values are the mean ± standard deviation (n = 18). The lowercase letters represent the significant differences (p < 0.05) between groups using the Duncan test.

To analyze the effects of HM application on maize cultivation, the effects of different fertilizers on plant growth were observed (**Figure 1**). The length of maize plants was considerably higher in the HM+CF group (285.34 cm) after 93 days than in the other groups (**Figure 1A**). However, there were no considerable differences in plant length between the CF (79.54 cm) and HM+CF (79.61 cm) groups after 51 days. The highest measured stem diameter and leaf length were in the HM+CF and CF groups in 51–93 days. On day 51, the leaf widths in different groups were in the order of HM+CF (6.76 cm), CF (6.51 cm), and HM (6.37 cm), while the HM+CF group had the largest leaf width (7.78 cm) at 93 days. In particular, plant length and leaf width increased in the HM+CF group compared to those in the CF group as the day progressed, suggesting a positive effect of HM in the late stages of plant growth. A high SPAD unit was observed in the HM+CF and CF groups after 51 days. The SPAD units of HM+CF and CF increased until 65 days and then decreased until 93 days, suggesting that the phase changed from vegetative to reproductive (**Figure 1B**). The highest SPAD units of the HM+CF group were observed after 65 and 79 days; no marked difference was observed between the SPAD units of HM+CF and CF groups after 93 days. The corn emergence rate after 79 and 93 days was highest in the HM+CF group and insignificantly different among the different experimental groups, except NT. The HM+CF and CF groups, which showed a relatively significant decrease in SPAD units, showed a higher emergence rate of corn after 79 days. Furthermore, the fresh and dry weights of corn were highly increased by HM+CF treatment. HM+CF and CF treatments improved plant growth compared to other treatments; in particular, HM+CF treatment demonstrated strong effects on plant length, leaf width, initial emergence rate, and corn weight. Although HM had a lower impact on the promotion of plant growth than the HM+CF and CF groups, the possible utilization of HM in agriculture needs to be considered.

### 3.3 Feed quality analysis and antioxidant activities

**Figure 2:**
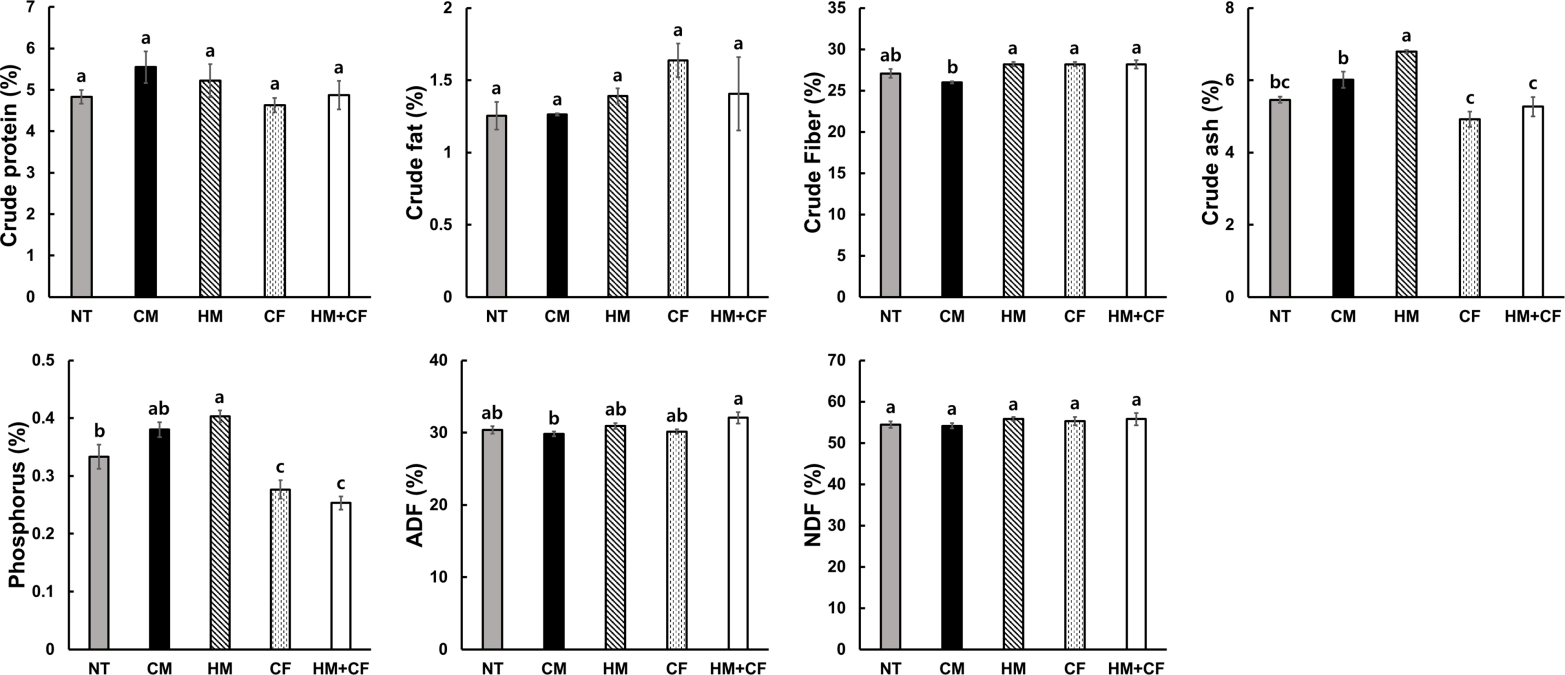
Feed values differ after applying different fertilizers (NT, non-treatment; CM, commercial manure; HM, Hanwoo manure; CF, chemical fertilizer; HM+CF, combined use of Hanwoo manure and chemical fertilizer). The values are the mean ± standard deviation (n = 3). The lowercase letters represent the significant differences (p < 0.05) between groups using the Duncan test.

**Figure 3:**
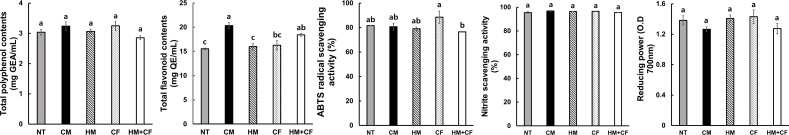
Total polyphenol and flavonoid contents, ABTS radical and nitrite scavenging activities, and reducing power activities after different fertilizer supplies (NT, non-treatment; CM, commercial manure; HM, Hanwoo manure; CF, chemical fertilizer; HM+CF, combined use of Hanwoo manure and chemical fertilizer). The values are the mean ± standard deviation (n = 3). The lowercase letters represent the significant differences (p < 0.05) between groups using the Duncan test.

To analyze the feed value of maize after supplying different fertilizers, we analyzed the contents of crude matter (protein, fat, fiber, and ash), P, ADF, and NDF in the plants (**Figure 2**). The crude contents of protein, fat, fiber, ADF, and NDF were not considerably different between the HM and CF groups. The crude ash and P contents were relatively high in the HM and CM groups, indicating that inorganic matter, including crude ash and P, increased in plants treated with organic manure. However, crude ash and P contents were low in the CF and HM+CF groups, indicating that inorganic matter in plants with CF supply was low. The contents and activities of antioxidants were analyzed in maize plants in terms of total polyphenols and flavonoids, 2,2’-Azino-bis (3-ethylbenzothiazoline-6-sulfonic acid) (ABTS), nitrite, and reducing power (**Figure 3**). The CM group had a relatively high total flavonoid content, and the HM+CF group had a relatively weak ABTS activity. No considerable difference was observed in the antioxidant levels of the HM and CF groups. In summary, the feed qualities, except for crude ash, P, and antioxidants, had a similar effect between the HM and CF groups.

### 3.4 Soil microbial composition

**Figure 4:**
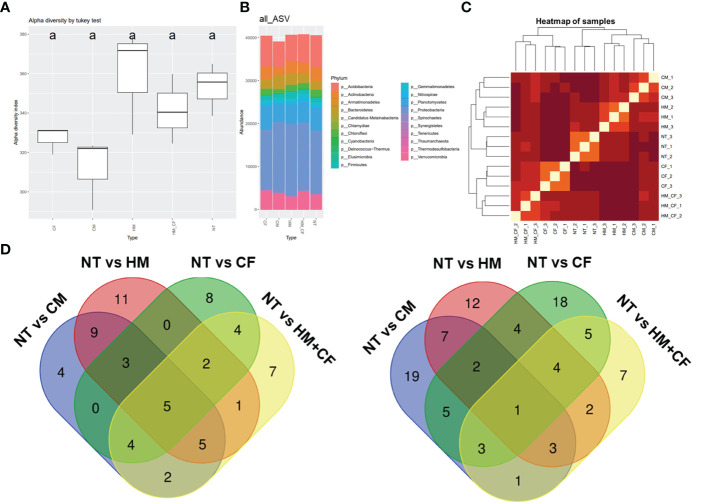
Amplicon sequence variant (ASV) approach of bacteria in the soils after different fertilizer supplies (NT, non-treatment; CM, commercial manure; HM, Hanwoo manure; CF, chemical fertilizer; HM+CF, combined use of Hanwoo manure and chemical fertilizer). **(A)** Alpha diversity of the bacterial population in each sample using the inverse Simpson (InvSimpson) method. The lowercase letters represent the significant differences (p < 0.05) between groups by using Tukey’s HSD test. **(B)** Bacterial communities of each sample in the phylum level. The box colors represent the phylum of bacteria. **(C)** Hierarchical clustering of samples. The line colors represent the different levels of beta diversity using the Bray−Curtis dissimilarity. **(D)** The comparison of differentially distributed bacteria in the soils after different fertilizer supplies.

To analyze the effects of different fertilizers on soil microbial communities, a 16s rRNA sequencing analysis was conducted (**Figure 4**). The HM group had a high alpha diversity (360 α-diversity) in the bacterial community. In comparison, the CF (327 α-diversity) and CM (312 α-diversity) groups had decreased alpha diversity compared to the NT (353 α-diversity) group (**Figure 4A**). However, there was no marked difference in the alpha diversity among the different soil samples. Soil microbial ASVs were clustered into 21 bacterial phyla (**Figure 4B**). Nine phyla (*Acidobacteria*, *Actinobacteria*, *Bacteroidetes*, *Chloroflexi*, *Firmicutes*, *Gemmatimonadetes*, *Planctomycetes*, *Proteobacteria*, and *Verrucomicrobia*) comprising over 1,000 ASVs were detected from soil bacteria in each sample. Notably, the ASVs of *Actidobacteria* and *Proteobacteria* constituted a large percentage of soil bacterial DNA (**Figure 4B**). The ASVs of *Acidobacteria*, *Actinobacteria*, and *Chloroflexi* were higher in the NT group than in the CM group (**Figure S2**). In contrast, ASVs of *Bacteroidetes* were high in the CM group and low in the NT group. Similarly, *Proteobacteria* and *Verrucomicrobia* were distinctly distributed between the HM and CF groups. High correlations of ASVs between soil samples were observed in two groups (CM and HM, and CF and HM+CF); in particular, HM+CF was closely correlated with CF (**Figure 4C**). This result suggests that the microbial community of the HM group was similar to that of the CF group, resulting in a change in the form of bacteria by supplying CF.

### 3.5 Associations between soil microbiome and fertilizer factors

To analyze differentially distributed bacteria, the ASVs of each soil sample were compared to those of the NT group at the phylum level (**Figure 4D**). In both soil samples, we obtained 158 considerable ASVs (65 increased and 93 decreased ASVs) divided into 21 phyla. The increased ASVs were 32 for the CM group, 36 for the HM group, 26 for the CF group, and 30 for the HM+CF group, whereas the decreased ASVs were 41, 35, 42, and 26 in the CM, HM, CF, and HM+CF groups, respectively. The highest number of significantly distributed bacteria was observed in the HM group for increased ASVs and in the CF group for decreased ASVs. This result suggests a positive effect of HM and a negative effect of CF on the bacterial community. Furthermore, many soil bacteria were commonly observed in CM, HM, CF, and HM+CF groups.

**Figure 5:**
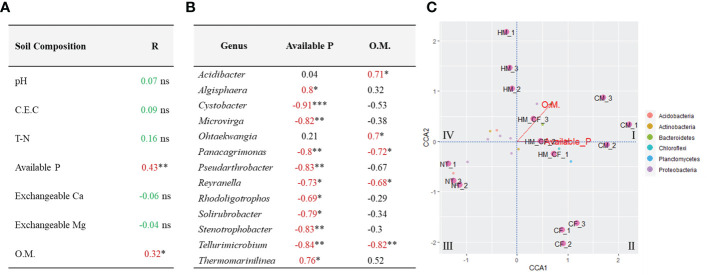
Correlation analysis between soil properties and bacterial community structures in the soils after different fertilizer supplies (NT, non-treatment; CM, commercial manure; HM, Hanwoo manure; CF, chemical fertilizer; HM+CF, combined use of Hanwoo manure and chemical fertilizer). **(A)** Mantel test between soil properties on bacterial community composition. Significant results are indicated by *P < 0.05 and **P < 0.01 (NS = no significance). **(B)** Significant correlation between two chemical components and soil bacteria. The significance was measured by using the n-2 degrees of freedom (*P < 0.05, **P < 0.01, and ***P < 0.001). **(C)** Canonical correspondence analysis of bacterial community composition and soil properties in soils after different fertilizer supplies.

**Figure 6:**
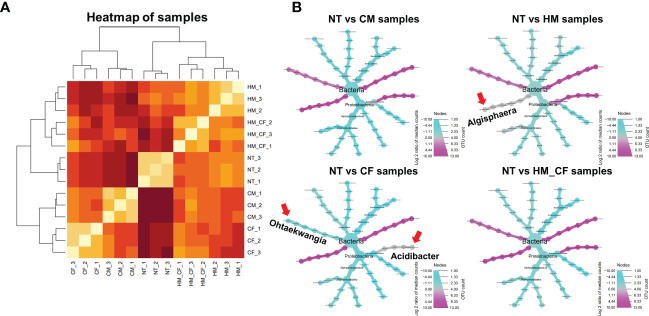
Taxonomic composition of significant bacteria analysis in the soils after different fertilizer supplies (NT, non-treatment; CM, commercial manure; HM, Hanwoo manure; CF, chemical fertilizer; HM+CF, combined use of Hanwoo manure and chemical fertilizer). **(A)** Hierarchical clustering of samples with significant bacteria. The line colors represent the different levels of beta diversity by using Bray−Curtis dissimilarity. **(B)** The relative abundances of significantly correlated bacteria in NT, CM, HM, CF, and HM+CF groups compared to the NT group. Circle size represents the number of bacteria for ASV. The color indicates the log 2-fold change of ASV with p-adjust < 0.05. Arrow represents the specific bacteria in each treatment.

We attempted to determine significantly correlated factors between the 158 ASVs and 7 soil chemical compositions, which had a significant difference in chemical quantities in at least one experimental soil across all samples (**Figure 5**). We found a significant positive correlation between available P (R = 0.43; p < 0.01) and OM (R = 0.32; p < 0.05) and bacterial communities (**Figure 5A**). Furthermore, we determined the significantly correlated bacteria (p < 0.05) for available P and OM within the bacterial phylum of 158 ASVs and found 13 significant bacterial genera (**Figure 5B**). *Algisphaera* (R = 0.8) and *Thermomarinilinea* (R = 0.76) were significantly positively correlated with available P content among the soil samples. In contrast, 9 bacterial genera (*Cystobacter*, *Microbirga*, *Panacagrimonas*, *Pseudarthrobacter*, *Reyranella*, *Rhodoligotrophos*, *Solirubrobacter*, *Stenotrophobacter*, and *Tellurimicrobium*) were negatively correlated with available P. OM was positively correlated with *Acidibacter* and *Ohtaekwangia* and negatively correlated with *Panacagrimonas*, *Reyranella*, and *Tellurimicrobium*. Three bacterial genera (*Panacagrimonas*, *Reyranella*, and *Tellurimicrobium*), which had negative correlations, were commonly exhibited in both P and OM. In the CCA analysis between chemical components and significantly correlated bacteria, OM and available P were closely correlated with livestock manures, except for NT and CF (**Figure 5C**). Although all bacterial ASVs were correlated between CF and HM+CF groups, the HM+CF group, along with livestock manure (HM and CM), was located close to OM and available P, as well as the significant bacterial genus (**Figure 5C**). This result suggests a distinct form of 13 significant bacterial genera in the HM+CF group due to increased OM and available P contents compared to those in the NT and CF groups. We identified a high correlation of 13 significant bacterial genera between the HM and HM+CF groups in the heatmap analysis, indicating a high distribution of specific bacteria in HM (**Figure 6A**) and specifically distributed bacteria in each experimental soil compared to NT (**Figure 6B**). *Ohtaekwangia* was found to be decreased in the CF group, while *Acidibacter* was increased in the other groups (CM, HM, and HM+CF groups), except in the CF group. *Algisphaera* increased in the CM, CF, and HM+CF groups, except for the HM group. The HM+CF group showed a distinct form of 13 significant bacteria between the HM and CF groups.

## 4 Discussion

In this study, we observed distinct phenotypic changes in plant growth for two years compared with the previous experiment by Byeon et al. (2022). There were no significant differences between the CF and HM+CF groups regarding OM content or plant growth. Notably, maize showed enhanced plant growth in the HM+CF group and increased OM content in the second year of the experiment, similar to the previous experiment. The combined supply of chemical and organic fertilizers positively improved crop production for long-term fertilization. The long-term use of organic fertilizers led to increased soil organic carbon (SOC) content compared to CFs (Wang et al., 2019). The combined supply of chicken manure and N increased the grain yield and dry matter content of spring maize compared with CF, suggesting that the appropriate proportion of organic substitution led to improved crop production (Geng et al., 2019). In a long-term fertilization experiment, Chand et al. (2006) demonstrated the beneficial effects of farmyard manure and CF in increasing dry matter and nutrient uptake for mint and mustard. In soil, nitrogen accumulation is stimulated in strongly acidic soils by organic manure application, along with swine manure and mushroom waste (Chen et al., 2001). Furthermore, Adekiya et al. (2020) reported that different sources of organic manure lead to increased amounts of OM, N, P, Ca, and Mg in the soil by enhancing the mineralization and steady supply of available nutrients. We identified relatively increased amounts of total N, exchangeable cations of Ca and Mg, and OM in the HM+CF group compared to the CF group, indicating soil nutrient changes caused by HM application. Thus, we suggest that the increased use of HM has additional effects on plant growth and soil nutrient changes.

Crude ash is related to the content of inorganic substances such as Ca, P, and Mg. In a previous study, the ash content in whole millet plants was increased by cattle manure and decreased by CF compared to NT; however, there were no significant differences in ash content among the different treatments (Raimundo et al., 2022). The crude ash content of onions was higher in the organic fertilizer-treated groups than in the control group without fertilization (Olle and Williams, 2014). Makinde et al. (2010) reported that organic material and its combination with CF caused a significant increase in crude ash content. Similarly, we identified increased contents of crude ash and P in the HM and CM groups, suggesting the positive effect of organic manures on the increase of inorganic matter in forage maize. Furthermore, there were no significant differences in antioxidant levels between the HM and CF groups. Thus, HM can replace CFs in the production of forage maize for livestock production.

We confirmed that 13 soil bacterial genera were significantly correlated with the OM and available P in the soil. The positively correlated bacteria were *Algisphaera* and *Thermomarinilinea* for available P and *Acidibacter* and *Ohtaekwangia* for OM. *Acidibacter* was a gram-negative bacterium isolated from a lake around a mine (Falagán and Johnson, 2014). The presence of *Acidibacter* is decreased by forest conversion because it prefers an acidic soil environment (Ezeokoli et al., 2020). In particular, *Ohtaekwangia* increased during the fermentation of sheep manure, and the presence of *Ohtaekwangia* and *Planomicrobium* could explain the suitability of sheep manure for using organic fertilizer (Yoon et al., 2011; Tortosa et al., 2017). We confirmed that *Ohtaekwangia* was significantly increased by organic manures, except for CF. *Algisphaera* had no significant differences in the HM group compared to the NT group, but it increased dramatically in the CM, CF, and HM+CF groups. *Algisphaera* is a gram-negative aerobic bacterium whose effects on plant growth remain unknown (Yoon et al., 2014). Furthermore, we found no significant difference in the presence of *Acidibacter* spp. in the CF group. *Acidibacter* was increased by organic manure application and correlated with SOC content (Guo et al., 2017; Jimenez-Castaneda et al., 2020; Zhang et al., 2021). Additionally, *Acidibacter* plays a role in the microbial reduction of Fe (III), and Fe (III) reduction by manure addition leads to an increase in pH (Falagán and Johnson, 2014; Jimenez-Castaneda et al., 2020). Thus, the enhanced microbial formation of *Acidibacter* by CM, HM, and HM+CF affected the pH of the soil.

## 5 Conclusions

The organic manure caused changes in soil nutrient compositions; in particular, the HM group showed increased amounts of total nitrogen, several exchangeable cations, and OMs compared to the CF group. The combined use of CF and HM led to improved plant growth compared with CF and organic manure. Although the HM+CF supply had substantial effects on plant growth, HM increased the inorganic matter content of crude ash, and P. ASV analysis revealed that increases in available P and OM drove 13 bacterial genera. We found specific bacteria, such as *Ohtaekwangia* and *Acidibacter*, in both organic manures, except for CF, and *Algisphaera* in others, except for HM. In particular, *Ohtaekwangia* and *Acidibacter* were considerably higher in organic manure. However, the beneficial interactions between significantly correlated bacteria and plants remain unknown. Further studies are needed to identify the functional roles of these soil bacteria in agriculture using organic manure. According to the results of this study, the use of HM as a useful organic source is suggested for environment-friendly agricultural practices, promoting sustainable crop production by replacing CFs.

## Data availability statement

The datasets presented in this study can be found in online repositories. The names of the repository/repositories and accession number(s) can be found below: BioProject, PRJNA923140.

## Author contributions

JL and S-GH conceived and designed the study. S-RK and M-GL provided the Hanwoo manure. JL, N-YJ, S-YS, LL, and S-GH led the field experiment. JL and S-GH performed ASV analysis. JL, N-YJ, S-YS, LL, and S-GH led the measurements of feed quality and antioxidant. All authors contributed to the article and critically to the drafts and revisions, and gave final approval for publication.
