# Supplementary figures and images for: Effects of Hanwoo (Korean cattle) manure as organic fertilizer on plant growth, feed quality, and soil bacterial community
Source: Front Plant Sci. 2023 Mar 21;14:1135947. doi: 10.3389/fpls.2023.1135947 (PMC10070840; doi:10.3389/fpls.2023.1135947)

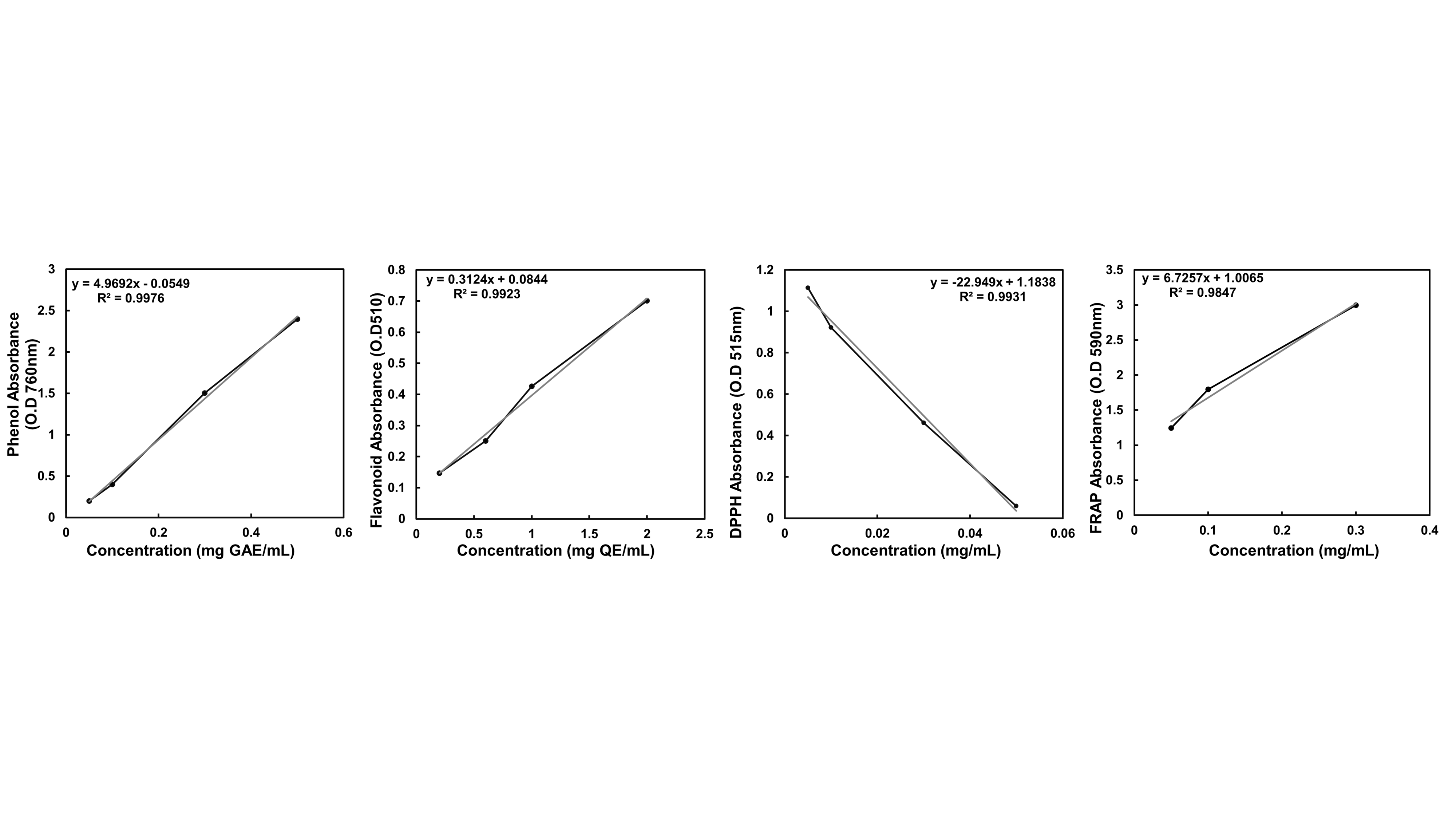

Supplement: Supplementary file 1 [file Image_1.tif]

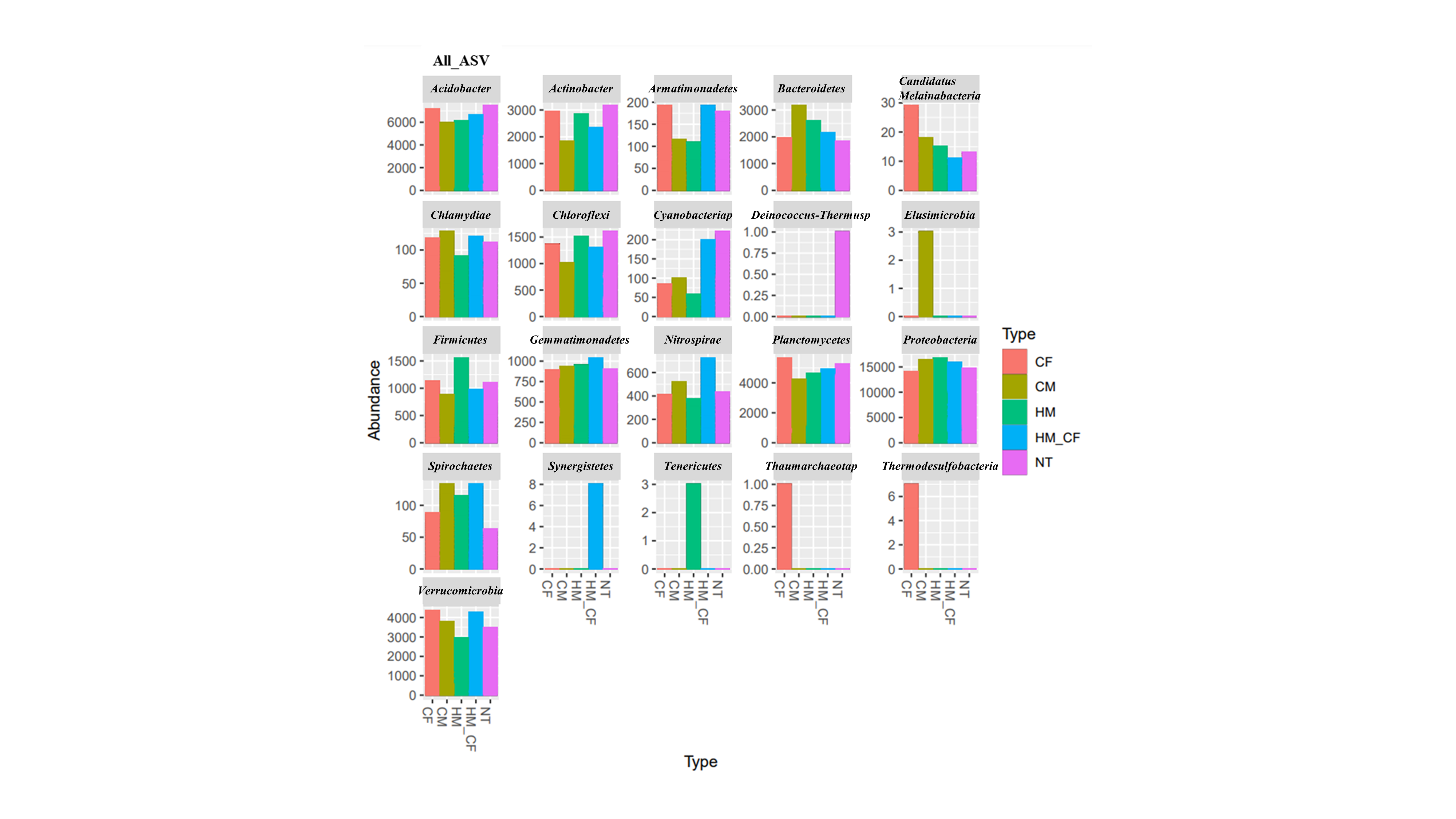

Supplement: Supplementary file 2 [file Image_2.tif]
